# Targeted elimination of senescent Ras-transformed cells by suppression of MEK/ERK pathway

**DOI:** 10.18632/aging.101325

**Published:** 2017-11-14

**Authors:** Elena Y. Kochetkova, Galina I. Blinova, Olga A. Bystrova, Marina G. Martynova, Valery A. Pospelov, Tatiana V. Pospelova

**Affiliations:** ^1^ Institute of Cytology, Russian Academy of Sciences, St-Petersburg, Russia

**Keywords:** senescence, autophagy, MEK/ERK, mitochondria, apoptosis

## Abstract

The Ras-Raf-MEK-ERK pathway plays a central role in tumorigenesis and is a target for anticancer therapy. The successful strategy based on the activation of cell death in Ras-expressing cells is associated with the suppression of kinases involved in Ras pathway. However, activation of cytoprotective autophagy overcomes antiproliferative effect of the inhibitors and develops drug resistance. We studied whether cellular senescence induced by HDAC inhibitor sodium butyrate in *E1a+cHa-Ras*-transformed rat embryo fibroblasts (ERas) and A549 human Ki-Ras mutated lung adenocarcinoma cells would enhance the tumor suppressor effect of MEK/ERK inhibition. Treatment of control ERas cells with PD0325901 for 24 hresults in mitochondria damage and apoptotic death of a part of cellular population. However, the activation of AMPK-dependent autophagy overcomes pro-apoptotic effects of MEK/ERK inhibitor and results in restoration of the mitochondria and rescue of viability. Senescent ERas cells do not develop cytoprotective autophagy upon inhibition of MEK/ERK pathway due to spatial dissociation of lysosomes and autophagosomes in the senescent cells. Senescent cells are unable to form the autophagolysosomes and to remove the damaged mitochondria resulting in apoptotic death. Our data show that suppression of MEK/ERK pathway in senescent cells provides a new strategy for elimination of Ras-expressing cells.

## INTRODUCTION

Cancer cells exploit a variety of protective mechanisms that allow them to acquire selective advantage and proliferate under unfavorable conditions. Macro-autophagy (hereafter referred to as autophagy) plays an important role in cancer cells survival upon various conditions of intra- and extracellular stress. Autophagy represents a caspase-independent process characterized by accumulation of autophagosomes in the cytoplasm and their subsequent fusion with lysosomes for degra-dation of unused or misfolded proteins or damaged structures such as ribosomes (ribophagy) and mito-chondria (mitophagy). Autophagy is an evolutionary conservative process that maintains cellular homeostasis and viability [1]. Autophagy can be either cytoprotective or cytotoxic in response to stress, chemotherapy or irradiation [2-4]. According to recent data, Ras-transformed cells require autophagy to survive and maintain malignant phenotype [5-10]. Therefore, autophagy is currently considered as a promising target for anticancer therapy that can also be successful in mutant Ras-expressing tumors charac-terized by chemotherapy resistance due to the protective autophagy.

Ras GTPase is frequently mutated in different types of cancers that correlate with a poor prognosis [11-15]. Despite a large number of promising inhibitors for Ras/Raf/MEK/ERK pathway, recent works show that cancer cells often develop an autophagy-dependent resistance to inhibitors of Ras pathway [16]. Oncogenic Ras induces the constitutive activation of Ras/Raf/MEK/ERK signaling pathway, which, in turn, activates other effector pathways, in particular, PI3K-mTOR signaling [17]. The catabolic processes regulated by mTORC1 control autophagy via a number of effector regulatory pathways [18, 19]. Given that autophagy is initiated upon suppressed mTORC1 activity, a protective autophagy in Ras-transformed cells with a high level of mTORC1 activity appears to be mTORC1-independent [20, 21]. One of the mechanisms of high basal autophagy activity in cancer Ras-expressing cells might be associated with high PP2A phosphatase activity targeting directly ULK1-Ser757 that could explain apparent contradiction: maintenance of high mTORC1 functions and high autophagic activity simultaneously [22]. This can provide Ras-expressing tumor cells additional selective advantages under stress or damage.

Playing a cytoprotective role in cancer cells, autophagy acts as an antagonist to such a powerful tumor-suppressor mechanism as cellular senescence, wherein cells permanently arrest their proliferation even in the presence of strong mitogenic signals [23-27]. Cancer cells expressing wild-type p53 can be induced to senescence by a variety of stimuli, including stresses, irradiation, starvation and histone deacetylase inhibitors (HDACi) [28-30]. Senescent cells do not proliferate but maintain viability and continue to synthesize proteins and to secrete the inflammatory cytokines and growth factors (SASP phenotype) [31, 32]. Interestingly, HDAC inhibitors can induce p53-independent cellular senescence in one type of cells and apoptosis in others [33].

In present work, we used rodent primary fibroblasts transformed by *E1Aad5* and *cHa-Ras* oncogenes (ERas cells) and A549 human lung adenocarcinoma cells that harbor Ki-Ras mutation. The ERas cells undergo HDACi-induced cellular senescence accompanied by cell hypertrophy and the activation of MEK/ERK pathway [34, 35]. A549 cells can also be induced to senescence [36]. Here, we aimed to study how HDACi-mediated cellular senescence would prevent appearance of resistance to MEK/ERK inhibitor PD0325901 (PD) and promote death of ERas cells. We showed that the suppression of MEK/ERK pathway in control ERas cells results in the damage of the internal structure of mitochondria and activation of AMPK-dependent cytoprotective autophagy that degrades the damaged mitochondria and thereby restores cell viability. In contrast, ERas cells induced to senescence do not develop a cytoprotective form of autophagy after inhibition of MEK/ERK pathway due to the spatial separation of lysosomes and autophagosomes in senescent cells that prevents their fusion and formation of autophagolysosomes. This leads to accumulation of the damaged mitochondria and an increase of caspase activity and ROS resulting in apoptotic cell death. Taken together, our data demonstrate that suppression of MEK/ERK pathway in ERas and A549 cells induced to senescence with HDACi provides a new successful strategy for elimination of *Ras*-expressing cells.

## RESULTS

### MEK/ERK inhibitor PD0325901 induces AMPK-dependent autophagy thus allowing ERas cells to develop resistance to MEK/ERK inhibitor and to restore their viability

**Figure 1:**
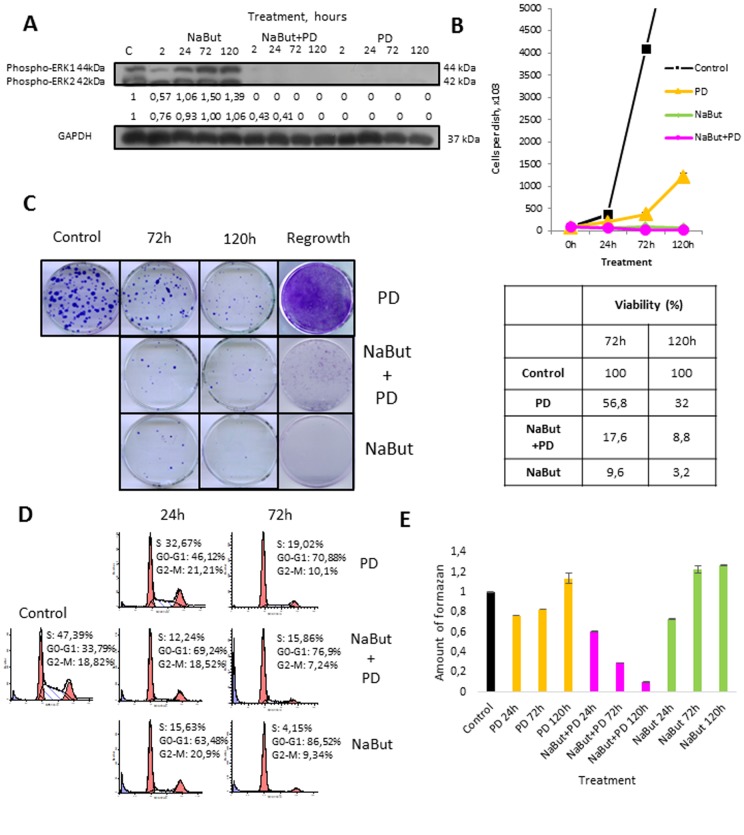
Autophagy promotes survival upon MEK/ERK inhibition in control ERas cells but cannot rescue senescent cells (**A**) Western-Blot analysis of ERK1,2 phosphorylation after short-term (2 h) and long-term (24 h-120 h) NaBut, NaBut+PD and PD treatment. Cells were cultivated with inhibitors for the indicated time and then lysed and processed to Western-blotting in 12% gel. Numbers below present densitometry of bands. (**B**) Growth curves of cells after exposure to inhibitors. The number of cells was counted after 24, 72 and 120 hours of experiment. Data are presented as mean ±S.E.M. of three independent replicates (n=3). (**C**) Clonogenic viability and proliferative potential of cells after removing the inhibitors. Cells were cultivated with inhibitors for 72 h and 120 h and then seeded at 200 cells per 30mm dish in drug-free medium. Clones were stained with Crystal Violet after 7 days of growth. Data are presented as mean ±S.E.M. of three independent replicates (n=3). For regrowth assay, cells were treated with inhibitors for indicated time and then provided with fresh inhibitor-free medium. Clones were stained Crystal violet after 5 days of growth in fresh media and counted. (**D**) Cell cycle distribution after exposure to inhibitors was analyzed by flow cytometry of propidium iodide-stained cells. Percentage of cells in G1, S and G2 phase indicated. (**E**) Viability was analyzed by MTT-test, amount of formazan was measured at 570 nm wavelength. Data are presented as mean ±S.E.M. of three independent experiments (n=3).

Here, we have used transformed rat embryo fibroblasts expressing *E1Aad5* and *cHa-Ras* oncogenes (ERas cells) as a model to study a role of MEK/ERK pathway in regulation of autophagy, which is involved in the maintenance of viability and implementation of senescence program. Senescence was induced by treatment with HDAC inhibitor sodium butyrate (NaBut, 4 mM). MEK1,2 inhibitor PD0325901 (PD, 1 μM) was used for long-term inhibition of MEK/ERK pathway. The treatment of ERas cells with PD0325901 leads to a complete cessation of ERK1,2 phosphoryla-tion that persists for 2-120 h as evidenced by Western-blot analysis (Fig. 1A).

According to cell growth assay and clonogenic survival data, PD0325901 treatment decreases proliferative activity of ERas cells, albeit the cell growth does not arrest to the full extent (Fig. 1B). The decrease of proliferation is most likely caused by inhibition of ERK1,2 phosphorylation involved in regulation of cell cycle progression [37]. Flow cytometry analysis reveals more than 2-fold decrease of cells in S-phase with simultaneous accumulation of cells in G1-phase (Fig. 1D). ERas cells decrease their viability after 24 h of PD0325901 treatment and then restore it as shown by MTT assay and this recovery is not associated with ERK1,2 phosphorylation (Fig. 1A). Cell proliferation is reactivated after providing the cells with fresh medium without inhibitor after 120h of treatment (Fig. 1C, E).

**Figure 2:**
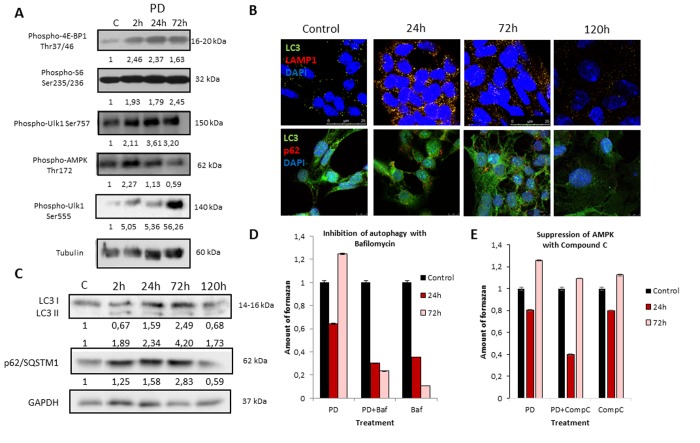
MEK/ERK suppression in control ERas cells leads to activation of AMPK-mediated autophagy which helps to overcome cell death (**A**) MEK/ERK suppression does not decrease mTORC1 activity and activates AMPK and Ulk1 Ser555 phosphorylation. Western-blot analysis of 4E-BP1, S6, AMPK, Ulk1 (Ser757, Ser555) phosphorylation after MEK/ERK suppression. Cells were treated with PD and then lysed and processed to Western-blotting in 10 and 12% gels. Numbers below present densitometry of bands. (**B**) Immunofluorescence images, demonstrating LC3 and LAMP1 colocalization and p62/SQSTM1 degradation. Cells were cultivated with PD, fixed and stained with antibodies against pan-LC3, LAMP1 and p62/SQSTM1 (p62). Confocal images are shown. Upper row: pan-LC3 (green), LAMP1 (red); bottom row: pan-LC3 (green), p62/SQSTM1 (red). Nuclei stained with DAPI (blue). Scale bars: 25μm (upper panel), 10 μm (lower panel). (**C**) Western-blot analysis of LC3I to LC3II conversion and p62/SQSTM1 degradation upon MEK/ERK suppression. Cells were exposed to PD and processed to Western-blotting in 15% gel. Numbers below present densitometry of bands. Decrease of viability of cells with inhibited MEK/ERK pathway after suppression of autophagy with Bafilomycin A1 (**D**) and after inhibition of AMPK with Compound C (**E**) as analyzed by MTT-test. Cells were treated with PD together with Bafilomycin A1 or with Compound C. In the indicated time intervals cells were processed with MTT reagent, amount of formazan was measured at 570 nm wavelength. Data are presented as mean ±S.E.M. of three independent experiments (n=3).

**Figure 3:**
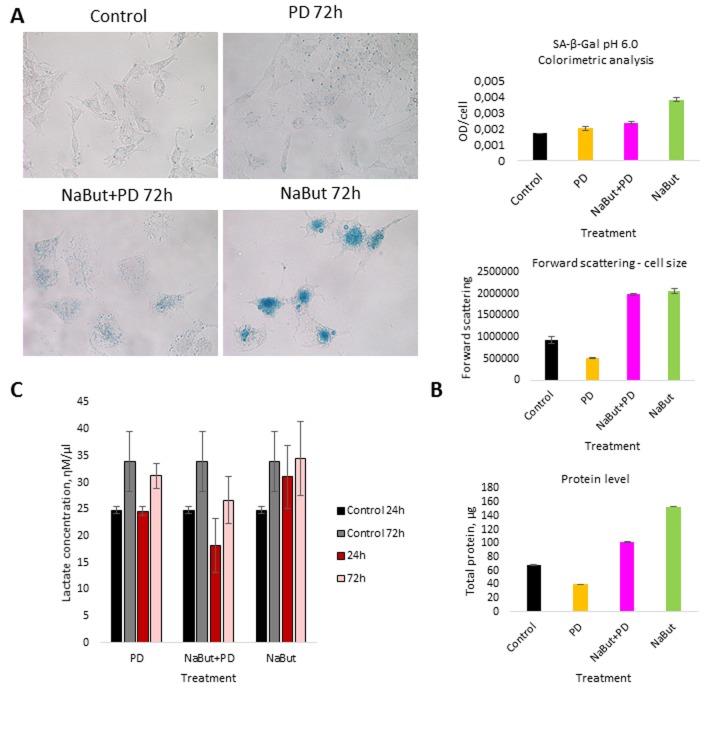
MEK/ERK suppression activates autophagy, which cancels the senescence program in control cells and attenuates senescence markers in NaBut-exposed cells (**A**) Senescence-associated β-galactosidase activity is high in senescent cells, but decreases upon MEK/ERK suppression. Visualized using Pascal LSM 5 microscope (images) and estimated by colorimetric assay (histogram). (**B**) Cell size and protein level decrease upon MEK/ERK suppression in intact cells and almost does not change in senescent cells where cytoprotective autophagy is disrupted. Cells were treated with inhibitors for 72 h. To evaluate cell size, cells were harvested and proceeded for flow cytometry to measure forward scattering. For protein content, 1×10^6^ cells were harvested, lysed and the protein amount was measured using Bradford assay. Data are presented as mean ±S.E.M. of three independent experiments (n=3). (**C**) Lactate level in culture medium decreases upon MEK/ERK suppression. Cells were treated with inhibitors for indicated time points, medium was collected and proceeded for measurement of lactate. Data are presented as mean ±S.E.M. of three independent experiments (n=3).

We further analyzed the role of autophagy in the development of resistance to MEK inhibition as well as in the restoration of viability and proliferation in long-term PD0325901 treated cells. It is well known that autophagy can be activated either by mTOR down regulation or AMPK activation [18-21]. We wondered how the autophagy could be affected upon MEK/ERK suppression by PD0325901. Although Ras-ERK pathway positively regulates mTORC1 by suppressing TSC2-RHEB [17], PD treatment did not lead to mTORC1 inhibition in control cells as shown by 4E-BP1 and S6 protein phosphorylation analysis (Fig. 2A). The level of Ulk1 Ser757 (the mTORC1 target) phosphorylation also did not decrease (Fig. 2A). Therefore, it appears more likely that mTORC1-independent autophagy is activated upon PD0325901 treatment. Then we assayed whether AMPK is activated in ERas cells treated with MEK inhibitor. Upon PD0325901 treatment, the level of AMPK phosphory-lation increases more than 2-fold at 2 h and 24 h of treatment (Fig. 2A). The level of pUlk1-Ser555, a pAMPK target responsible for the initiation of autophagy [20, 21], also increases throughout 2-72 h of treatment. The dynamics of pAMPK and pUlk1-Ser555 accumulation coincides with the activation of autophagy markers during PD0325901 treatment (LC3-I to LC3-II conversion, p62/SQSTM1 accumulation (Fig. 2B). Thus, PD0325901 induces the AMPK-dependent autophagy in ERas cells, despite the high level of mTORC1 activity, which is a potential negative regulator of autophagy. Given that a key regulator of cellular senescence, mTORC1, is active in PD0325901 treated cells, while cell cycle is blocked, we aimed to analyze the levels of senescence markers. The obtained results show that in ERas cells treated with PD0325901 for 72 h the total protein level and the cell size are slightly decreased (Fig. 3B). In addition, they do not demonstrate senescence-associated β-galactosidase (SA-β-gal) activity (Fig. 3A). Thus, despite the persisting block of the cell cycle and especially the high activity of mTORC1, the suppression of MEK/ERK activates autophagy, which abolishes the development of senescence program. The prolonged suppression of MEK/ERK activity was shown to reduce p38 MAP kinase phosphorylation [38]. As p38 MAPK is involved into regulation of replicative and premature senescence [39], we assessed the levels of p38 phosphorylation in control and senescent cells upon MEK/ERK suppression. According to Western blot data, the level of phospho-rylated p38 decreases in both control and senescent cells upon PD0325901 treatment, while senescent cells with active MEK/ERK pathway have a higher level of p38 MAPK phosphorylation (Supplementary Fig. 1). Thus, a decrease of p38 activity in PD-treated ERas cells might contribute to downregulation of senescence markers. Recently, it was shown that inhibition of p38 phospho-rylation can promote autophagy [40].

We then assessed autophagic flux by immuno-fluorescent staining and Western-blot analysis. Immunofluorescent and Western blot data show that PD0325901 treatment activates autophagy as follows from LC3-I to LC3-II conversion and colocalization of LC3 with a lysosomal marker LAMP1 at 24-72 h time interval (Fig. 2B,C). Another marker of autophagy – an autophagy cargo receptor p62/SQSTM1 protein, which degrades after fusion of autophagosomes with lysosomes, is accumulated to 72 h of PD0325901 treat-ment, but then disappears at 120 h correlating well with Western-blot data of LC3 I-II conversion (Fig. 2B, C). We infer that autophagy flux increases in a period of 24-72 h and then returns to a control level at 120 h. This results correlate with the restoration of cell viability and proliferative activity as assessed by a clonogenic assay and analysis of cell growth of PD0325901-treated cells after removal of inhibitor (Fig. 1C, E). Suppression of autophagy with Bafilomycin A1 decreases the viability of control ERas cells that indicates the ERas-transformed cells require autophagy to maintain their viability. Co-treatment of cells with PD0325901 and Bafilomycin A1 results in a significant decrease of cellular viability compared to cells treated with PD alone, where cellular viability restores 72 h post treatment, thereby suggesting a cytoprotective role of autophagy activation in control ERas cells (Fig. 2D). To clarify whether the development of PD0325901 resistance is linked to AMPK-mediated autophagy, we used an ATP-competitive inhibitor of AMP-kinase activity Compound C together in combination with PD0325901. We found that exposure of PD-treated cells to Compound C decreased cell viability compared to PD0325901 alone (Fig. 2E). Thus, these data support a conclusion that AMPK is involved in autophagic response of ERas cells to MEK/ERK suppression.

**Figure 4:**
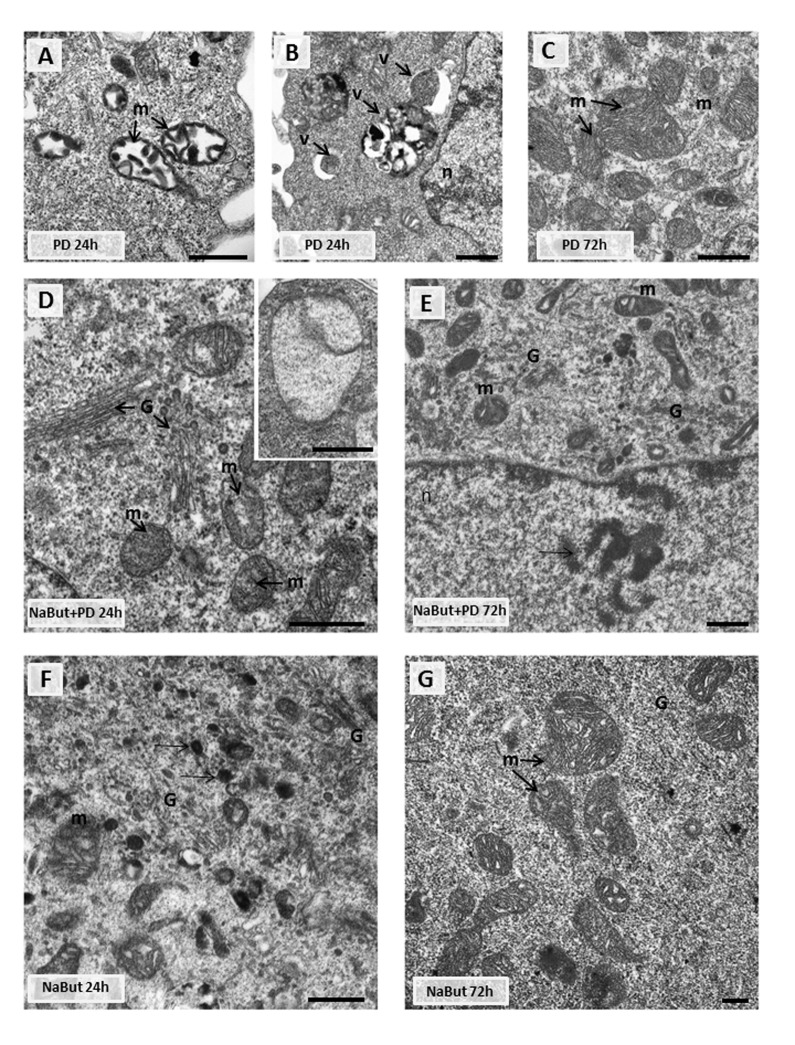
Transmission electron microscopy (TEM) images showing the ultrastructure of intact and senescent ERas cells treated with inhibitor of MEK/ERK-pathway (A, B, C) Representative images of the mitochondria in intact ERas cells after MEK/ERK suppression. Note severe alterations of mitochondria (m) after 24 hours of treatment (**A**), mitochondria in autophagosome-like vesicles (**B**) and normal-looking mitochondria after 72 hours of treatment (**C**). (**D**) Representative image of senescent ERas cell treated with PD for 24 hours. It contains well-developed Golgi apparatus (G) and mitochondria (m) with rare cristae. Inset: mitochondria with complete loss of the cristae and the preserved double-membrane envelope. (**E**) Representative image of senescent ERas cell treated with PD for 72 hours. The cell contains well-developed Golgi apparatus and swollen mitochondria with vacuolar structure. Note poorly developed nucleoli (*arrow*). The senescent ERas cells after 24 (**F**) and 72 (**G**) hours of NaBut exposure. Stacks of Golgi cisternae and lysosome-like structures (F, *arrows*) can be seen. Designations: G, Golgi; m, mitochondria; n, nucleus; v, vesicle. Scale bars: 500 nm.

**Figure 5:**
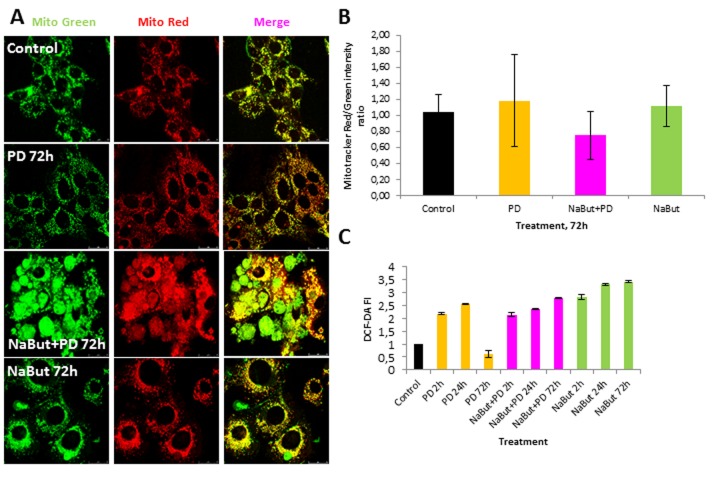
Suppression of MEK/ERK pathway in senescent cells leads to accumulation of damaged mitochondria and reactive oxygen species (ROS) (**A**) Damaged mitochondria accumulate in senescent cells with suppressed MEK/ERK (according to Mitotracker Green and Mitotracker Red *in vivo* staining). At indicated time points cells were stained with Mitotracker Red and Mitotracker Green and images were acquired using Leica TSC SP5 microscope. (**B**) Graphical representation of Mitotracker Red/Mitotracker Green intensity ratio in control and treated cells. ImageJ software was used to analyze the images. (**C**) ROS accumulate in senescent cells with suppressed MEK/ERK after 72 h of cultivation. At indicated time points cells were incubated with DCF-DA and fluorescence was measured at proper wavelength. Data are presented as mean ±S.E.M. of three independent replicates (n=3).

Given that mitochondria ensure the viability of Ras-expressing cells and autophagy is required to maintain the pool of functional mitochondria [6, 7, 10], we analyzed the integrity of mitochondria using transmission electron microscopy (TEM) and immuno-fluorescent staining with Mitotracker Red (potential-dependent) and Mitotracker Green (potential-independent). TEM data demonstrate that mitochondria have severe ultrastructural alterations in ERas cells treated with PD0325901 for 24 h. They are vacuolated and have disrupted cristae (Fig. 4A). The damaged mitochondria can be observed in numerous auto-phagosome-like vacuoles (Fig. 4A, B), indicating that they are degraded presumably by mitophagy. After 72 h of PD0325901 treatment the ultrastructure of mitochondria does not differ from control cells, and the damaged mitochondria are no longer detected (Fig. 4C). In addition, both Mitotracker Red and Mitotracker Green fluorescent signals are active in ERas cells treated with PD0325901 for 72 h. Mitotracker Red/Mitotracker Green intensity ratio is not decreased, indicating that the mitochondria retain their membrane potential (Fig. 5A, B). Mitochondria damage is associat-ed with the increased levels of reactive oxygen species (ROS) at 2 h and 24 h of MEK/ERK suppression in senescent cells, as follows from an assay with DCF-DA, which is oxidized by ROS into 2′, 7′–dichloro-fluorescein (DCF). DCF is a highly fluorescent compound which can be detected by fluorescence spectroscopy with maximum excitation and emission spectra of 495 nm and 529 nm respectively. However, ROS levels in cells with suppressed MEK/ERK do not exceed those in control cells as damaged mitochondria are no longer present (Fig. 5C). We infer that the effects caused in ERas cells by PD0325901 treatment are at least due to mitochondria damage. However, after the activation of AMPK-mediated autophagy in long-term PD0325901 treated cells the viability of cell population restores. Thus, ERas cells respond to MEK/ERK inhibition by activating AMPK-mediated autophagy, which promotes recovery of mitochondrial integrity and cellular survival.

### Inhibition of MEK/ERK pathway in ERas cells induced to senescence with NaBut impairs autophagy and cellular survival

When cell cycle is blocked, the high level mTORC1 favors the protein synthesis and cell hypertrophy, maintains energy homeostasis, senescence-associated secretory phenotype (SASP) and viability [23, 25, 41]. However, in control Ras-transformed cells elevated activity of mTORC1 provides viability and supports high proliferation rate. Therefore, we asked how MEK/ERK inhibition by PD0325901 affects the viability of cells induced to senescence with NaBut and whether PD0325901 is able to induce AMPK-dependent autophagy in the senescent cells.

First, we analyzed how PD0325901 treatment affects viability of NaBut-treated senescent ERas cells. Growth curves and clonogenic survival data show that MEK-ERK inhibition in cells induced to senescence leads to almost 10-fold suppression of viability as compared with control ERas cells. Similar data follows from the MTT viability assay (Fig. 1E). NaBut induces an irreversible cell cycle arrest and suppression of proliferation in ERas cells resulting in senescence [34, 42, 43], however the arrested cells remain viable as confirmed by MTT assay (Fig. 2E). The flow cytometry data reveal a sub-diploid peak upon NaBut and PD0325901co-treatment implying the apoptotic cell death (Fig. 2D).

**Figure 6:**
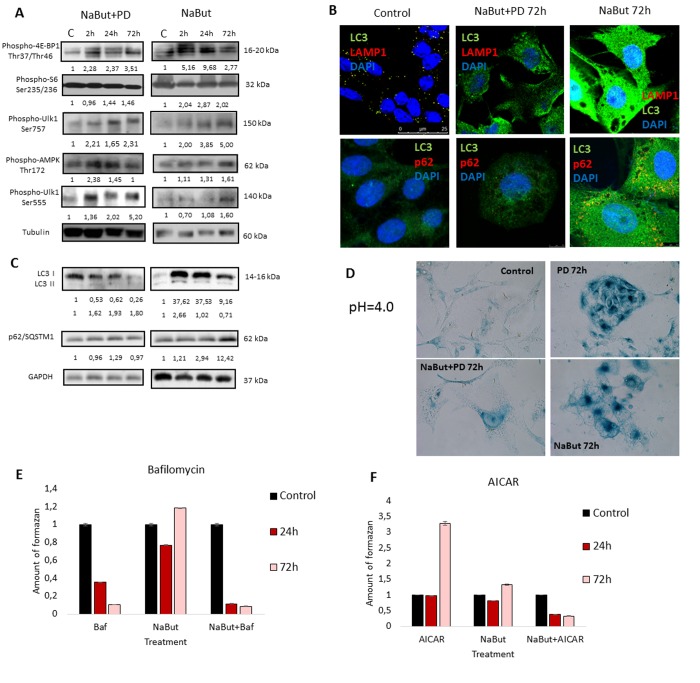
Senescent ERas cells fail to induce cytoprotective autophagy upon MEK/ERK inhibition (**A**) Senescent cells with suppressed MEK/ERK retain high mTORC1 activity and phosphorylate AMPK and Ulk1 Ser555. Cells were treated with NaBut+PD or NaBut and then lysed and processed to Western-blotting in 10 and 12% gels. Numbers below present densitometry of bands. (**B**) Immunofluorescence images, demonstrating that senescent cells with suppressed MEK/ERK have no LC3 and LAMP1 colocalization. Cells were treated with inhibitors or left untreated, fixed and stained with antibodies against pan-LC3, LAMP1 and p62/SQSTM1 (p62). Confocal images are shown. Upper row: pan-LC3 (green), LAMP1 (red); bottom row: pan-LC3 (green), p62/SQSTM1 (red). Nuclei stained with DAPI (blue). Scale bars: 25 μm (upper panel), 10 μm (lower panel). (**C**) Western-blot analysis of LC3I to LC3II conversion and p62/SQSTM1 degradation. Cells were processed to Western-blotting in 15% gel. Numbers below present densitometry of bands. (**D**) Images demonstrating low lysosomal β-galactosidase activity in senescent cells with MEK/ERK suppression. Cells were treated with inhibitors for 72 h and then fixed and stained with β-galactosidase substrate in pH 4.0 buffer and visualized using Pascal LSM5 microscope. Decrease of viability of senescent cells treated with autophagy inhibitor Bafilomycin A1 (**E**) or AMPK mimetic AICAR (F), analyzed by MTT-test. Cells were treated with NaBut and Bafilomycin A1 or NaBut and AICAR. Cells were processed with MTT reagent, amount of formazan was measured at 570 nm wavelength. Data are presented as mean ±S.E.M. of three independent experiments (n=3).

**Figure 7:**
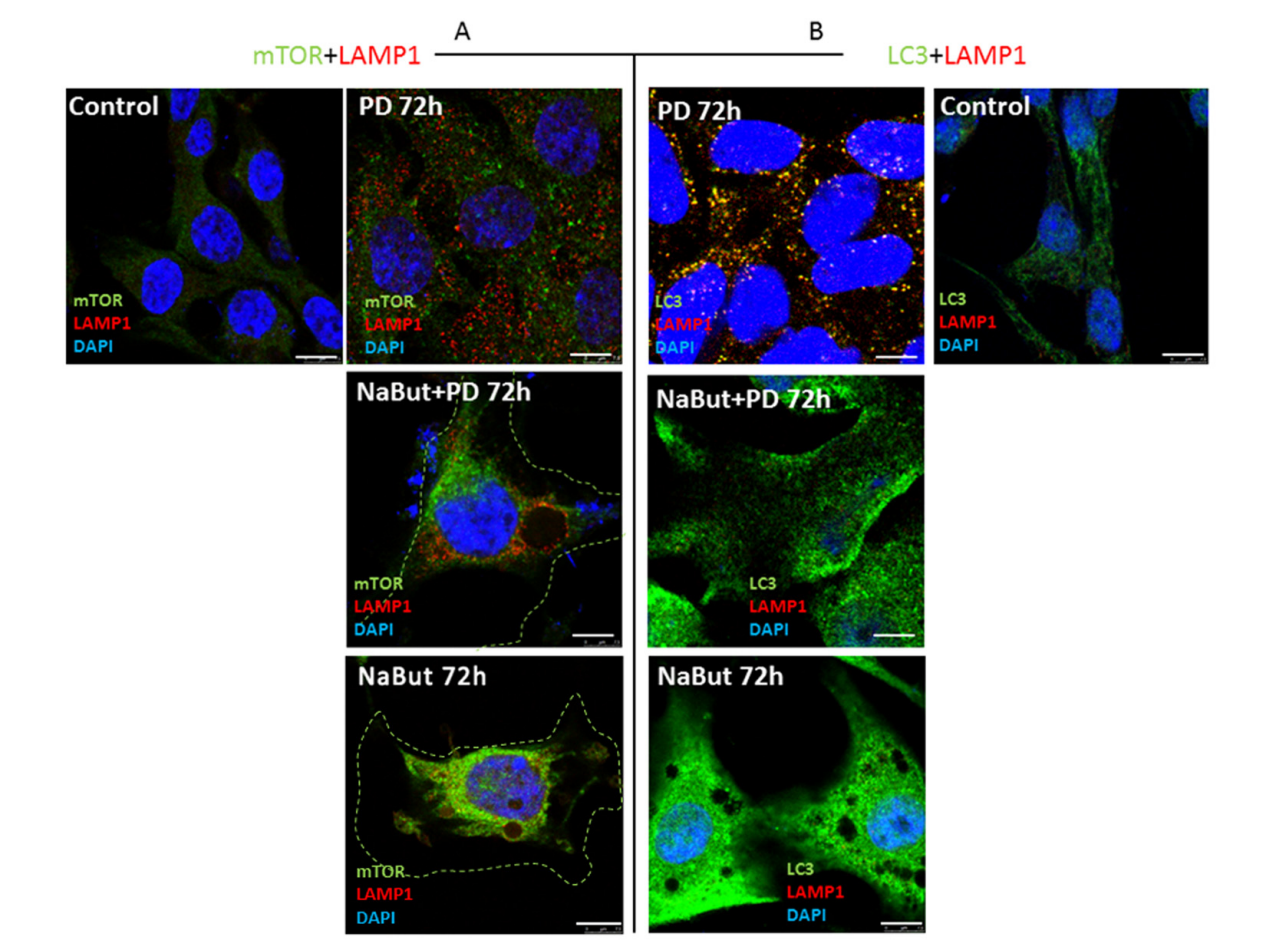
Spatial uncoupling of lysosomes and autophagosomes in senescent cells blocks their fusion upon activation of autophagy by MEK-ERK inhibition Immunofluorescent images showing LAMP1 (red) and mTOR (green) perinuclear localization in senescent cells and in senescent cells upon MEK/ERK suppression. Cells were treated with inhibitors for 72 h, then fixed and stained with antibodies against LAMP1/mTOR (**A**) and LAMP1/LC3 (**B**). Green dotted lines show borders of senescent cells (**A**). Right panel (**B**) shows the actual size of senescent cells according to LC3 fluorescence. LAMP1 colocalizes with LC3 in control cells exposed to PD and does not colocalize in senescent cells. Nuclei stained with DAPI (blue). Scale bars: 7,5 μm.

Next, we investigated whether PD0325901 activates AMPK-regulated autophagy in cells induced to senescence by NaBut. Senescent cells treated with PD0325901 accumulate phosphorylated AMPK at 2 h and 24 h of treatment followed by accumulation of phosphorylated autophagy-responsible target pUlk1-Ser555 (Atg1) (Fig. 6A). However, the fusion of autophagosomes with lysosomes is completely blocked as judged by the lack of colocalization of the LC3 and LAMP1 (Fig. 6B, 7B). Both PD0325901- and NaBut-treated cells demonstrate elevated lysosomal β-galactosidase levels (pH 4.0), a marker of the increased lysosomal activity, that localizes predominantly around the nuclei (Fig. 6D). However senescent cells with suppressed MEK/ERK signaling also demonstrate peri-nuclear lysosomal β-galactosidase (pH 4.0) localization, but intensity of staining is weaker indicating the lower lysosomal activity. Thus, we suggest that in senescent cells exposed to MEK/ERK inhibitor the activity of lysosomes and autophagosome-lysosome fusion are disrupted. It was previously shown that in senescent cells lysosomes predominantly co-localize with mTOR [44]. Our data indicate that NaBut-treated cells (72 h) demonstrate colocalization of mTOR and LAMP1 in perinuclear regions, while upon MEK/ERK suppression LAMP1 does not colocalize with mTOR (Fig.7A). Besides, senescent cells with suppressed MEK/ERK activity have a weaker LAMP1 signals that are not detected at the periphery of the cytoplasm, where LC3 is mostly localized. These cells also do not demonstrate LAMP1 and LC3 colocalization, as can be seen by immunofluorescent staining data (Figs. 6B and 7B). Low level of LC3-II revealed at 2 h of PD treatment as assessed by Western blotting then disappears conco-mitantly with a decrease of the overall cytoplasmic level of LC3 (Fig. 6B, C). Analysis of p62/SQSTM1 immuno-fluorescence staining shows neither accumulation nor its colocalization with LC3 (Fig. 6B, C). Together, this data show that senescent cells with suppressed MEK/ERK pathway are characterized by defective autophagy that fails to support viability of senescent cells. To clarify whether AMPK activation is cytotoxic for these cells, we used AICAR, a compound that is converted to monophosphorylated nucleotide (ZMP) within the cell, thus mimicking a decrease of ATP and hence activating ATP synthesis through AMPK activity. Treatment of senescent ERas cells with AICAR resulted in a significant decrease of cellular viability, while in control cells AICAR increased viability after 72 h of treatment according to MTT assay (Fig. 6E). These data indicate that, while in control cells AMPK activity favors viability, in senescent cells even early steps of AMPK activation are cytotoxic.

We have shown that inhibition of MEK/ERK signaling by PD0325901 initially leads to mitochondrial damage, but then cells can restore the mitochondrial function and proliferative potential. We asked whether the damaged mitochondria also degrade in senescent ERas cells treated with PD0325901. TEM data shows that senes-cent ERas cells treated for 24 h with PD0325901 have the mitochondria with disordered cristae and electron dense or concentric membrane structures inside the organelles as well as with partial to complete loss of the cristae. The mitochondria devoid of cristae are present as the rounded electron-empty structures with double membranes (Fig. 4D,E). Although the mitochondria are severely disordered, they are not localized within the autophagosomes in contrast to the control ERas cell treated with PD alone (Fig. 4B). Thus, the electron microscopy and immunofluorescence data indicate the absence of autophagosome-lysosome fusion and mitophagy activation in senescent cells. Therefore, senescent cells are not capable of removing damaged mitochondria. Furthermore, cells co-treated with NaBut and PD0325901 for 24 h have the enlarged Golgi complexes with the numerous stacks of flattened cisternae surrounded by multiple vesicles. Considering the well-developed nucleoli, all together indicates that the ongoing active protein synthesis associates with a high level of mTORC1 activity. Senescent ERas cells exposed to PD0325901 for 72 h demonstrate the abundant of damaged mitochondria with swollen cristae and hyperdense matrix (Fig. 4E). Accordingly, senescent cells with suppressed MEK/ERK show the increased levels of ROS caused by the mitochondria damage. ROS levels remain high up to 72 h of treatment, unlike control cells treated with PD0325901. Analysis of the main markers of senescence in NaBut and PD0325901 co-treated cells shows a high level of senescence-associated SA-β-gal activity (pH 6.0) and increased mTORC1 activity in addition to cell cycle arrest and cell hypertrophy. However, these parameters are slightly lower than in NaBut-treated senescent cells (Fig. 3A, B). Despite high mTORC1 activity, the Golgi complexes are numerous, but the nucleoli demonstrate weak development at 72 h of NaBut and PD0325901 co-treatment (Fig. 4E). ERas cells treated with NaBut alone for 24 h and 72 h show numerous stacks of Golgi cisternae and lysosome-like structures (Fig. 4). Mito-chondria retain their usual structure in senescent cells (Fig. 4F, G). Interestingly, NaBut-treated cells have increased ROS level (Fig. 5C) that can be explained by senescence activation rather than the mitochondrial damage [45, 46].

The tumor cells are characterized by distinctive features such as high proliferative rate and glycolysis as a predominant source of ATP production [47]. Our data demonstrate that the suppression of MEK/ERK pathway in senescent cells does not abolish mTORC1 activity, specific for NaBut-treated cells. It is well known that mTORC1 is involved in up-regulation of glycolysis [48- 50]. When mitochondria are damaged, the cells can activate glycolytic activity to maintain viability, thereby surviving the unfavorable conditions until the mitochon-dria will be restored [51]. Therefore, we checked whether glycolysis is indeed affected in senescence cells treated with PD0325901 by measuring the lactate level in culture medium. The results indicate that suppression of MEK/ERK in senescent cells reduces the production of lactate, while senescent cells show a much higher level of lactate production (Fig. 3C). Similarly, control cells treated with PD0325901 alone for 72 h have lower levels of lactate compared to control cells. We suggest that PD decreases the lactate levels due to the involvement of MEK/ERK pathway in regulation of several glycolytic genes [52-54]. Accordingly, senescent cells with suppressed MEK/ERK pathway are characterized by the lowest levels of lactate at 72 h of treatment, thus confirming that glycolysis in this case is suppressed. We believe that inhibition of glycolysis together with the disturbance of mitochondrial function contributes to cell death of PD0325901-treated senescent cells.

**Figure 8:**
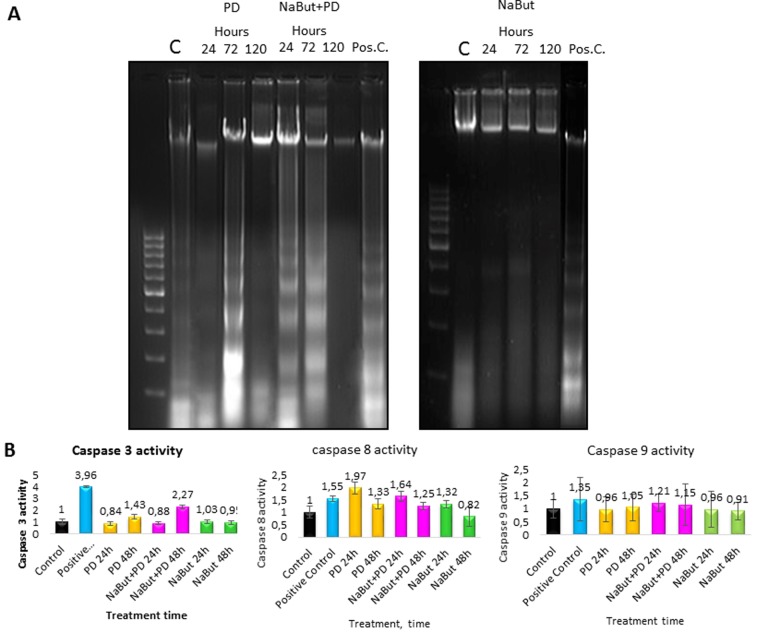
ERas cells undergo caspase 3- and caspase 8-regulated apoptosis upon MEK/ERK inhibition ERas cells undergo caspase 3- and caspase 8-regulated apoptosis upon MEK/ERK inhibition. (**A**) 2% agarose gel-electrophoresis of DNA fragments extracted from cells after exposure to inhibitors shows apoptotic nucleosomal DNA fragmentation in senescent cells with suppressed MEK/ERK. Cells were treated with inhibitors for indicated time or left untreated and processed to electrophoresis. DNA visualized with ethidium bromide. DNA fragments extracted from serum-starved ERas cells undergoing apoptosis were used as a positive control. (**B**) Graphical representation of data on caspase 3, 9 and 8 activities after treatment with inhibitors. At the indicated times caspase 3 and 8 activities were measured using Ac-DEVD-AMC for caspase-3, Ac-VETD-AMC for caspase 8 and Ac-LEHD-AFC for caspase 9 fluorogenic substrates at proper wavelengths. Data are presented as mean ±S.E.M. of three independent replicates (n=3).

A direct evidence of the activated apoptotic program in senescent ERas cells with repressed MEK/ERK pathway follows from analysis of the nucleosomal DNA fragmentation. As shown by DNA fragmentation assay in 2% agarose gel, inhibition of MEK/ERK in cells undergoing NaBut-induced senescence results in the nucleosomal DNA fragmentation after 24 h and 72 h of treatment (Fig. 8A). According to a clonogenic assay, PD0325901 alone also induces apoptotic cell death at 72 h of treatment, while about 50% of cells in population remain viable (Fig. 1C). The obtained results are in accordance with a 2-fold activation of caspase-3 in NaBut and PD0325901 co-treated cells as compared to control ones, and a 1,5-fold increase in case of PD0325901 treatment alone (Fig. 8B). Interestingly, an increased activity of caspase-8 but not of caspase-9 can be observed in NaBut and PD0325901 co-treated cells.

Thus, inhibition of MEK-ERK pathway in NaBut-treated senescent cells interferes with the autophagy programs which serve to support a stringent balance of catabolic and anabolic processes in senescent cells provided by mTORC1. Due to a defective autophagy, the cells are incapable of maintaining senescence program and viability. Unlike the control cells exposed to PD0325901 alone, the senescent cells are unable to eliminate the damaged mitochondria thus giving rise to accumulation of the defected mitochondria and resulting in apoptotic cell death.

### NaBut and PD0325901 co-treatment causes redistribution of Ras from the plasma membrane to the cytosol and its co-localization with LC3

**Figure 9:**
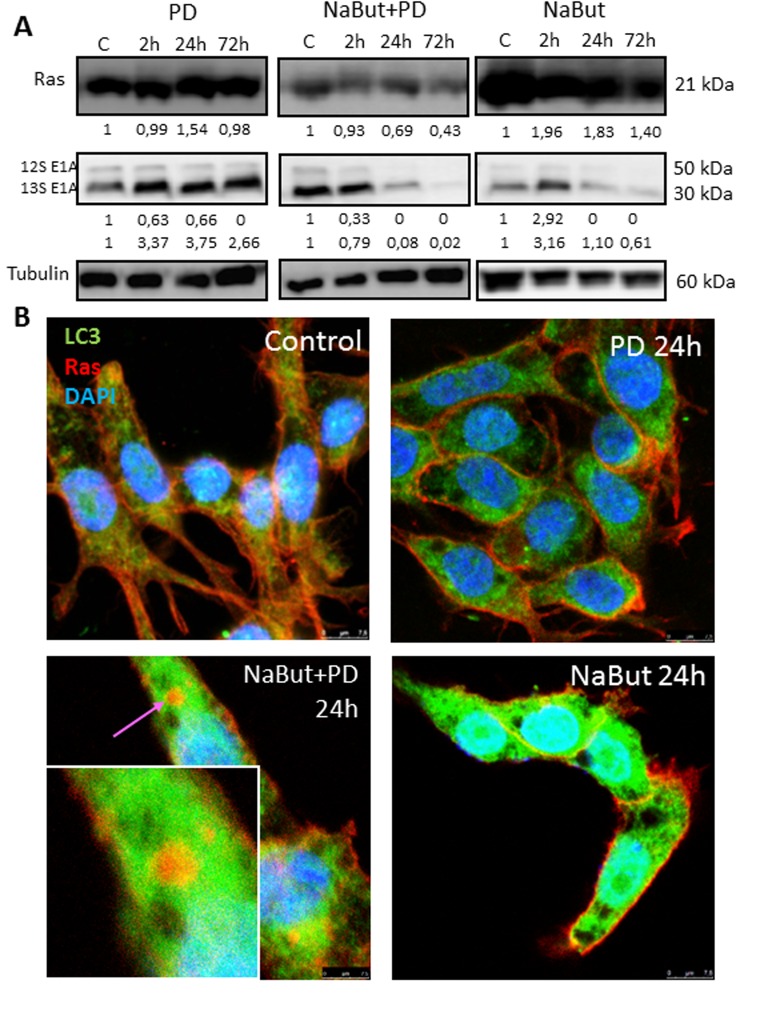
MEK/ERK suppression in senescent ERas cells changes balance of E1A and Ha-Ras oncoproteins and leads to relocalization of Ras from the plasma membrane into the cytoplasm (**A**) Western-blotting analysis of Ras and E1A (12S, 13S) expression. Cells were exposed to inhibitors and processed to Western-blotting in 12% gel. Numbers below present densitometry of bands. (**B**) Immunofluorescent images show changes of Ras (red) and LC3 (green) localization in senescent cells with suppressed MEK/ERK. Cells were treated with inhibitors for the indicated time, then fixed and stained with antibodies against pan-Ras and pan-LC3. Square indicates a magnified region showing Ras in the cytoplasm colocalized with LC3 in senescent PD-treated cell. Nuclei stained with DAPI (blue). Scale bars: 25 μm.

Oncogenic Ras provides both pro-autophagic and anti-apoptotic functions within the cell [55, 56]. It is shown that autophagy provides survival of Ras-expressing tumor cells by maintenance of the mitochondrial function [6, 7, 10]. We showed that the inhibition of MEK/ERK pathway in senescent ERas cells leads to accumulation of damaged mitochondria due to defects of autophagy. We wondered whether the oncogenic Ras and E1A content is a subject of change due to autophagy during PD0325901 treatment of ERas senescent cells. We wanted to know whether the expression and localization of anti-apoptotic Ras onco-protein, which is also a key regulator of autophagy and cell viability, can change in ERas cells treated with PD0325901, NaBut or both inhibitors together. Western blot data show that unlike treatment with PD0325901 or NaBut alone, inhibition of MEK/ERK in senescent cells causes a decrease in the Ras content (Fig. 9A). Similarly, the expression of complementing E1A oncoprotein did not decrease in PD0325901 treated cells. However, overall amount of E1A decreas-ed in NaBut-treated ERas cells with increasing time and then significantly downregulated with development of senescence.

The most significant changes occur in the colocalization of the Ras protein. While in untreated control, in PD0325901-treated and in senescent ERas cells the Ras protein is localized under plasma membrane (Fig. 9B), no membrane-bound Ras has been detected in senescent cells upon 72 h of PD0325901 treatment. Immunofluorescence staining using antibodies against Ras and LC3 indicates that Ras colocalizes with LC3 in the cytoplasm of cells co-treated with NaBut and PD0325901 for 24 h. Thus, Ras re-localizes from the membranes to the cytoplasmic structures in senescent cells upon PD0325901 treatment, where it most possibly fails to function as a regulator of signal transduction and autophagy. Thus, in senescent cells PD0325901 does not only inhibit the MER/ERK pathway, but also promotes the oncogenic Ras re-localization, thereby disrupting its function as an autophagy regulator in senescent ERas cells [7, 8].

**Figure 10:**
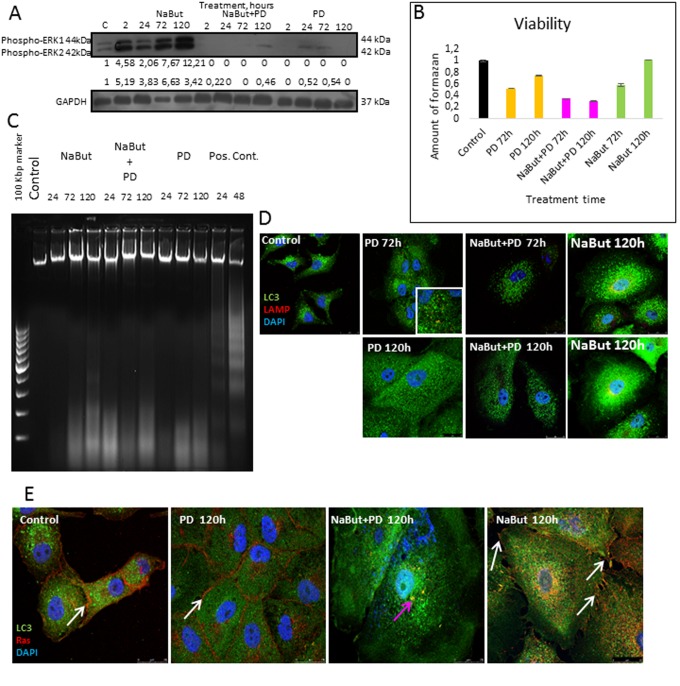
Senescent human Ki-Ras-mutated lung adenocarcinoma A549 cells after MEK/ERK suppression undergo autophagy impairment, Ras relocalization and cell death (**A**) Western-blot analysis of ERK1,2 phosphorylation after short-term (2 h) and long-term (24 h-120 h) NaBut, NaBut + PD and PD treatment. Cells were exposed to inhibitors for the indicated time and then lysed and processed to Western-blotting in 12% gel. Numbers below present densitometry of bands. (**B**) Cell viability analyzed by MTT-test. In the indicated time intervals cells were processed with MTT reagent, the amount of formazan was measured at 570 nm wavelength. Data are presented as mean ±S.E.M. of three independent experiments (n=3). (**C**) Electrophoresis in 2% agarose gel of DNA extracted from untreated cells and cells after exposure to inhibitors shows the absence of nucleosomal DNA fragmentation in treated cells. DNA visualized with ethidium bromide. DNA extracted from serum-starved ERas cells undergoing apoptosis was used as a positive control. (**D**) Immunofluorescence images demonstrating LC3 and LAMP1 levels and colocalization after 72 h and 120 h of treatment. Cells were treated with inhibitors or left untreated and stained with antibodies against pan-LC3 (green) and LAMP1 (red). Nuclei stained with DAPI (blue). Scale bars: 25 μm. (**E**) Immunofluorescent images showing Ras (red) and LC3 (green) localization after 120 h of treatment. Cells were exposed to inhibitors for indicated time, then fixed and stained with antibodies against pan-Ras and pan-LC3. *White arrows* show membrane-bound Ras; *pink arrow* shows Ras in cytoplasm of senescent cells with inhibited MEK/ERK. Nuclei stained with DAPI (blue). Scale bars: 25 μm.

To clarify whether the cell response to MEK/ERK inhibition by PD0325901 is specific for the ERas-engineered cell line or inhibition of MEK/ERK signaling would successfully eliminate also other Ras-expressing senescent tumor cells, we used Ki-Ras mutated A549 human lung adenocarcinoma cells. Inhibition of MEK/ERK pathway in A549 cells simultaneously with induction of senescence by NaBut leads to a decrease of cell viability at 72-120 h of treatment (Fig. 10B). Сells undergo non-apoptotic death according to nucleosomal DNA fragmentation analysis (Fig. 10C). According to immunofluorescent staining data, A549 cells show colocalization of LAMP1 and LC3 at 72 h upon MEK/ERK suppression, whereas no LAMP1 signal has been detected 120 h after treatment (Fig. 10D). Our data indicate that MEK/ERK suppression in control A549 cell results in the induction of autophagy that lasts for 72 h. A pronounced LAMP1 staining can be detected in senescent A549 cells nearby the nuclei at 120 h post treatment. In addition, LAMP1 colocalizes with LC3 upon 120 h treatment. However, senescent A549 cells with suppressed MEK/ERK pathway show very weak LAMP1 signal at 120 h of treatment, and no LAMP1-LC3 colocalization can be detected in these cells (Fig. 10D). These data allow us to conclude that in senescent A549 cells lysosomal activity and autophagy are disrupted by PD0325901 treatment. Also, A549 cells induced to senescence by NaBut and treated with PD0325901 demonstrate Ras co-localization with LC3 in the cytoplasm, implying that this phenomenon may be common for various Ras-expressing cells (Fig. 10E).

## DISCUSSION

Here we showed that *E1A+cHa-Ras*-transformed embryo fibroblasts respond to MEK/ERK suppression by activating AMPK-regulated autophagy. These data are consistent with the recent results obtained in other Ras-expressing tumor cells such as MDA-MB-231 and HT29 [57]. Correspondingly, suppression of autophagy was shown to reduce viability and clonogenic survival of Ras-mutated Calu3, H322C, HCC4006 and H2009 cell lines [58]. Nevertheless, several works performed on M229 R5, M238 R1 and M14 melanoma cells and on Ki-Ras-mutated colorectal cancer cell lines show that these cells are able to acquire the resistance to MEK/ERK suppression [59]. Restoration of ERK1,2 phosphorylation by activation of PI3K signaling pathway indicate that elimination of Ras-mutated cancer cells seems to be more complicated process [60]. Nevertheless, despite the lack of ERK1,2 phosphory-lation recovery, ERas cells can restore viability and proliferation upon MEK/ERK suppression. Thus, an alternative pathway(s) provides restoration of ERas cells proliferation. These pathways may involve ERK5 signaling as was shown for the intestinal tumor cells [61]. The authors found that in genetically engineered mice with ERK1,2 deficiency, proliferation of intestinal tumor cells was rescued by activation of ERK5 signaling. We found out that rodent ERas cells and human lung adenocarcinoma A549 cells also restore their viability in the absence of recovery ERK1,2 phosphorylation upon PD0325901 treatment, suggest-ing another pathway for restoration of cell viability, which is not unique for intestinal ERK1,2 deficient cells.

One may suggest that the cellular response to MEK/ERK suppression involves not only AMPK, but also other pathways, most probably PI3K/AKT signal-ing pathway [62]. Despite the complete absence of phospho-ERK1,2 upon PD0325901 treatment, ERas cells retain active mTORC1. Of note, Ras-ERK and PI3K-mTOR pathways form a negative feedback loop in relation to each other, thereby suppression of one pathway leads to the activation of another [62]. Thus, suppression of MEK1,2 in a subset of breast cancer cell lines results in the EGFR mediated activation of PI3K/AKT pathway [63]. According to our results, no significant increase of pAKT-Ser473 phosphorylation has been observed in response to PD0325901 treatment (Supplementary Fig. 1), therefore, this negative loop is not functional in ERas transformed cells.

Despite the high activity of mTORC1 and cell cycle arrest the PD0325901 treated ERas cells do not undergo senescence due to the activation of AMPK-dependent autophagy that blocks the senescence program. A number of reports indicate that a proper activation of autophagy is required for the establishment of senescence [64-67], while others show that autophagy suppresses senescence [68, 69]. In case of PD0325901-treated ERas cells induced to senescence by NaBut, the latter scenario may take place, because the forced autophagy could lead to the isolation and degradation of proteins important for the senescence establishment. It has been shown that inhibition of mTORC1 by rapamycin and Torin1 in IMR90 ER:RAS senescent human cells reduces the expression of senescence marker SA-β-gal and development of SASP without rescuing cells from the proliferation arrest [70]. In addition to cell cycle arrest, high mTORC1 activity is necessary for the establishment of senescence [23, 25, 71, 72]. Although in NaBut and PD0325901 co-treated cells these requirements are fulfilled, the senescence is attenuated and cells undergo apoptosis. Cells activate tumor suppressor programs such as apoptosis or cellular senescence in response to injury or stress. It has also been reported that senescent cells are resistant to apoptosis [73-75]. The suppression of MEK/ERK pathway in NaBut-senescent ERas cells with high TORC1 activity initially induces mTORC1-independent autophagy that destabilizes stringent anabolic and catabolic equilibrium of senescent cells resulting in apoptosis.

We have shown that exposure of senescent ERas cells to MEK/ERK inhibitor leads to accumulation of damaged mitochondria and their subsequent degrada-tion by a mitophagy-like process resulting in the absence of damaged mitochondria 72 h after treatment. However, suppression of MEK/ERK signaling in senescent cells causes accumulation of the damaged mitochondria. Several reports suggest that the activity of ERK1,2 kinases and mitochondria functioning are connected. Particularly, in response to the suppression of ERK1,2 phosphorylation a decrease of ATP production and mitochondria damage can be observed in alveolar macrophages [76]. Moreover, for such tumor cells as prostate cancer RWPE-2 and DU145 cells and osteosarcoma Saos-2 cells was shown that mito-chondrial fraction of active ERK1,2 kinases provides resistance to apoptosis inducers by desensitizing cells to mitochondrial permeability transition pore [77]. Authors have shown that the ERK1,2 kinases act through GSK3-Cyclophilin D axis, and application of MEK-ERK inhibitors significantly increased mitochondrial depolarization and cell death in response to apoptosis inducers. To sum up, these data show that phospho-rylation of mitochondrial ERK1,2 plays an important role in the maintenance of mitochondrial integrity and ATP levels. Thus, MEK/ERK inhibition could lead to loss of ATP levels as well as provide vulnerability of mitochondria, thus triggering AMPK activation and mitophagy that is involved in elimination of damaged mitochondria.

Interestingly, tumor cells with damaged mitochondria can maintain their viability by enhancing glycolysis [51]. Respectively, a decrease of glycolysis is fatal for the cell as exemplified by our results on lactate assay in senescent cells treated with PD0325901. The emerging defects of autophagy in senescent ERas cells with suppressed MEK/ERK pathway reduce the level of glycolysis and promote the increased sensitivity to stress and result in apoptosis.

Therefore, a question arises why inhibition of MEK/ERK pathway in senescent cells does not activate a functional cytoprotective autophagy capable of degrading the damaged mitochondria? The answer may be found in the structural and functional organization of the cytoplasm of senescent cells. The increasing number of data demonstrate that spatio-temporal organization of the cytoplasm plays an essential role in implementing regulation of cellular senescence. Thus, the lysosomes in senescent cells associate with mTOR to form so-called TOR-autophagy spatial coupling compartments (TASCC), which are localized near the endoplasmic reticulum and Golgi complex [44]. In addition, the TASCCs were found to be devoid of autophagosomes [78].

We found that in senescent ERas cells the fusion of autophagosomes and lysosomes is entirely blocked as seen from the lack of LC3 and LAMP1 co-localization. The reason for this may be linked to the fact that these structures are spatially separated. We found that LAMP1 is localized near the nucleus, whereas LC3 is detected mainly on the periphery of the cytoplasm. Unlike the senescent cells treated with NaBut alone, LAMP1 is spatially separated from mTOR in senescent PD-treated cells, indicating that the strict com-partmentalization required for maintaining the senescent phenotype is altered in this case. Senescent cells have lysosomes tethered predominantly nearby nucleus, similarly to the conditions of nutrient starvation [79], while autophagosomes are at the periphery of the cell. For nutrient starvation it was shown, that mTORC1 is activated after moving to the periphery with the lysosomes, while being localized near nucleus it is inactive [79], though it is unclear yet, through what mechanisms lysosome positioning affects mTORC1 activity. Senescent NaBut-treated cells exhibit active mTOR located nearby nucleus, lysosomes are also located in perinuclear region, autophagosomes at the periphery, so the level of autophagosome-lysosome fusion is very low, in requirement to support homeo-stasis and preserve the hypertrophy. Another reason for the incapability of senescent ERas cells to develop a correct autophagic response after MEK/ERK inhibition can be explained by relocalization of oncogenic Ras from plasma membranes to the cytoplasm. Normally, Ras is a membrane-associated protein and its proper localization is essential both for maintaining the functional activity and for the interaction with the downstream target signaling proteins, especially those that regulate autophagy [80]. Re-localization of Ras into autophagosomes will greatly weaken its both anti-apoptotic and pro-autophagic functions, thus changing the cellular response to damage.

In addition, it was demonstrated that in human fibroblasts induced to senescence by various factors, including stress, replicative exhaustion and oncogene over-expression, mTORC1 is constitutively activated, and the senescent cells are resistant to serum deprivation and amino acid starvation [81]. The resistance partly develops due to autophagy, which provides the cells with a sufficient level of amino acids, thereby supporting high mTORC1 activity upon deprivation. However, persistent mTORC1 activity prevents senescent cells from the development of full autophagic program that is detrimental and leads to cell death [81] or attenuation of cellular senescence [70, 82]. It is appropriate to add that re-localization of oncogenic Ras from the plasma membrane to the cytoplasm coincides in time with accumulation of damaged mitochondria.

In summary, we show that suppression of MEK/ERK pathway in ERas-transformed cells induced to senescence is more destructive than in control cells, due to the apoptotic cell death, associated with the accumulation of damaged mitochondria. The damaged mitochondria are not eliminated by autophagy in the senescent cells thus providing accumulation of ROS and eventually resulting in cell death. The сo-treatment with MEK/ERK inhibitor PD0325901 and senescence inducer NaBut is applicable not only for elimination of ERas rodent cells, but also for human Ki-Ras-expressing A549 cells. Our data give a new insight into regulation of autophagy, metabolic and energy balance and the maintenance of viability in senescent cells. Besides, the results can impact the development of new strategy for elimination of Ras-expressing tumor cells.

## MATERIALS AND METHODS

### Cell culture and treatment

The work has been performed using rat embryonic fibroblasts, co-transformed with *E1A* region of Ad5 DNA and human *cHa-Ras* oncogene (Q12 and G61 mutated), and human non-small lung adenocarcinoma A549 cells. Cells were cultured in DMEM with 10% FBS (HyClone), penicillin and streptomycin in 5% CO2 at 37 ^°^C. Histone deacetylase inhibitor sodium butyrate (NaBut, 4 mM, Sigma) was used to induce senescence. PD0325901 (PD, 1 μM, Sigma) was used to inhibit MEK1,2 activity and ERK1,2 phosphorylation. Compound C (2 μM, Calbiochem, CAS 866405-64-3) and AICAR (Calbiochem, CAS 2627-69-2) were used to determine the role of AMPK in cellular response to NaBut and PD0325901. Bafilomycin A1 (Calbiochem, 196000-10UG, 10 nM) was used to inhibit autophagy in senescent or PD0325901 treated cells.

### Cell proliferation analysis

Cells were seeded at the initial density of 3×10^4^ cells per 35 mm cell culture dish (Costar, USA) in 3 repeats 24 h before the treatment. Cells were treated with NaBut and PD0325901 or left untreated. The medium was replaced by a fresh one supplemented with 10% FBS and inhibitors every second day. The number of cells was daily counted in the cell counting chambers throughout 120 h. The growth curves were plot based on the data obtained in 3 independent experiments.

### Flow cytometry

Cells were seeded at initial density 15×10^4^ per 60 mm cell culture dish (Costar, USA) and treated with inhibitors for indicated time. Cells were harvested in phosphate-buffered solution, permeabilized with 0,01% saponin and incubated with 100 μg/ml RNAse A and 40μg/ml propidium iodide for 15 min at 37°C. Cell cycle distribution was analyzed using Odam (Brucker, France) flow cytometer.

### Cell viability assay

For clonogenic survival assay, cells were seeded at initial density 3×10^4^ cells per 35 mm cell culture dish and treated with inhibitors for 72 and 120 h. At the indicated time points, cells were trypsinized and plated at the density of 200 cells per 35 mm cell culture dish in fresh, inhibitor-free medium. After 7 days of cultiva-tion, colonies were stained with Crystal Violet counted. For regrowth assay, cells were treated with inhibitors for 120 h, then medium was changed for inhibitor-free one and cells were left to grow. After 120 h, dishes were stained with Crystal Violet. For MTT assay, cells were seeded at 12-well plate at initial density 2×10^4^ cells per well and treated with inhibitors for indicated time. Then medium was replaced with 0,5 mg/ml MTT reagent solution and cells were incubated for 1 h at 37°C. After 1 h, MTT solution was replaced with DMSO to dissolve formazan, and cells were incubated for 30 min. Amount of formazan was measured at 572 nm wavelength, using DMSO as a blank solution. The resulting optical densities were normalized to optical density of untreated control cells, measured in 24 h after seeding.

### Senescence-associated β-galactosidase detection

SA-β-Gal expression was analyzed as previously described [83]. Images were acquired using LSM5 Pascal microscope (Carl Zeiss Microscopy). Colori-metric analysis was performed at 595 nm wavelength and normalized to cell number.

### Lysosomal activity

Lysosomal β-galactosidase was analyzed as previously described [83], using acetate/phosphate buffer adjusted to pH 4.0. Images were acquired using LSM 5 Pascal microscope (Carl Zeiss Microscopy).

### Protein content and cell size

Cells were treated with inhibitors for 72 h, then 1×105 cells were collected and lysed in RIPA buffer. Protein concentration was measured using Bradford method. To evaluate cell size, cells were treated with inhibitors for 72 h, then harvested and subjected to forward light scattering analysis using Odam (Brucker, France) flow cytometer.

### Analysis of lactate level in culture medium

Cells were grown in 12-well plates in triplicates and treated with inhibitors. At the indicated time points medium was collected, centrifuged using Amicon filters (Sigma-Aldrich, Z740170-8EA), and lactate was measured using a BioVision kit (BioVision, #K607-100) as described by manufacturer.

### ROS levels

Cell were treated with inhibitors for the indicated time points and then incubated with 10 μM 2′-7′-dichlorodihydrofluorescene diacetate (DCF-DA, Invitrogen D399) for 30 min. DCF fluorescence was measured using FLUOstar Omega (BMG Labtech) microplate reader and normalized to cell number.

### DNA fragmentation assay

Cells were lysed in a buffer containing 10 mM Tris-HCl (pH 8,0), 0,1M EDTA (pH 8,0) and 0,5% SDS for 20 min at 4°C. Then samples were centrifuged and NaCl (0,15 M) and RNAse A (100 μg/ml) were added to supernatant. The probes were incubated for 1 h at 37° С. Then samples were incubated with 0,5% SDS and 200 μg/ml Proteinase K and incubated for 2 h at 37° С. DNA was deproteinized by a mixture of water-saturated phenol pH 8.0/chloroform. DNA was reprecipitated, washed with 70% of ethanol, and dried. For electrophoresis, DNA was dissolved in 1 mM TE buffer pH 8.0 and subjected to electrophoresis in 2% agarose gel (Sigma). DNA bands were stained with ethidium bromide. DNA isolated from ERas cells starved for 24 h in 0.5 % FCS undergoing apoptosis [84] was used as a positive control.

### Western-blotting

Cells were lysed in RIPA buffer (1% IGEPAL, 0,5% Sodium deoxycholate; 0,1% SDS; 50 mM TRIS-HCl pH 8,0; 150 mM sodium chloride; 5 mM EDTA pH 8,0; 60 mM sodium fluoride) supplemented with the Roche Protease inhibitor cocktail. 50 μg of protein were separated by SDS-PAGE electrophoresis in 15%, 12% or 10% gels and transferred to PVDF membranes (0,2 μM, EMD Millipore). Membranes were blocked with 5% fat-free milk (Sigma) for 1 h following the incubation with primary antibodies in TBS solution containing 3% BSA and 0,1% Tween-20 overnight at 4°C. The membranes were washed and incubated with secondary antibodies conjugated with horseradish peroxidase. Blots were developed by enhanced chemi-luminescence (ECL, ThermoFisher Scientific). GelPro3 Software (Media Cybernetics) was used to perform densitometry.

### Immunofluorescence

Cells were seeded at coverslips at initial density 5×10^4^ cells per 35 mm cell culture dish and treated with inhibitors at the indicated time points. Cells were fixed, permeabilized with 0,2% TRITON X100, blocked in 3% BSA in TBS-T, and incubated in primary antibodies overnight at +4°C following the incubation with secondary antibodies for how long and what tempe-rature. Coverslips were mounted with DAPI-containing ProLong Gold mounting medium (Molecular Probes, P36931). Cells were visualized using Leica TSC SP5 microscope (Leica Microsystems).

### Caspase activity assay

Cells were lysed in Lysis buffer (50 mM HEPES-HCl, pH 7,4; 0,5% IGEPAL; 0,1% CHAPS; 5 mM DTT) and incubated for 30 min at 4°C. The protein concentration was measured and 50 μg of protein was incubated for 1h at 37°C in the dark with with Reaction buffer (20 mM HEPES-HCL, pH 7,4; 0,1% CHAPS; 5 mM DTT) containing fluorogenic substrates: Ac-DEVD-AMC (EMD Millipore) for caspase-3, Ac-VETD-AMC (Sigma) for caspase-8 and Ac- LEHD-AFC (EMD Millipore) for caspase-9. Serum-starved ERas cells were used as a positive control [84]. Fluorescence was measured at following wavelengths: Ex/Em=380/460 nm for caspase 3 substrate, 435/538 nm for caspase 8 substrate, 400/505 nm for caspase 9 substrate.

### Mitotracker Red and Mitotracker Green staining for detection of mitochondrial integrity

Cells were seeded on coverslips and treated with the inhibitors for indicated time. Then cells were stained with Mitotracker Red and Mitotracker Green (Invitrogen, M7513 and M7514, respectively) as described by manufacturer. The intensity of fluorescence was visua-lized using Leica TCS SP5 microscope (Leica Micro-systems) at following wavelengths: Ex/Em=577/599 nm for Mitotracker Red, 490/516 nm for Mitotracker Green. Images were analyzed using ImageJ software.

### Transmission electron microscopy

For transmission electron microscopy, cells were seeded on coverslips and treated with inhibitors, fixed for 1 h at 4°C in 2,5% glutaraldehyde in 0.1 M cacodylate buffer (pH 7.3), containing 0,15 M sucrose, postfixed for 1 h in 1% osmium tetroxide in the cacodylate buffer, dehydrated and embedded in a mixture of Epon and Araldit. Ultrathin sections were cut using a diamond knife on a LKB ultratome, collected on fine mesh copper grids, and stained with uranyl acetate and lead citrate for the examination with Zeiss Libra 120 electron microscope operated at 80 kV.

### Statistical analysis

Data are presented as means of three independent experiments; statistical significance was evaluated by Student's t test, where P<0,05 was considered statistically significant.

### Antibodies

The following primary antibodies were used: pan-LC3 (MBL, #PM036); p62/SQSTM1 (BD Transduction, #610077); LAMP1 (Santa-Cruz, sc-17768); pan-Ras (Oncogene Science, #OP40); E1A (Santa-Cruz, sc-58658); phospho-Ulk1 Ser757 (Cell Signaling, #6888S); phospho-Ulk1 Ser555 (EMD Millipore, ABC124); phospho-AMPK T172 (Cell Signaling, #2535S); phospho-4E-BP1 Thr37/46 (Cell Signaling, #2855S); phospho-S6 Ser235/236 (Cell Signaling, #2211S); phospho-42/44 MAPK Thr202/Tyr204 (Cell Signaling, #4377S); phospho-Akt Ser473 (Cell Signaling, #4060S); phospho-p38 MAPK Thr180/Tyr182 (Cell Signalling, #9211); mTOR (Cell Signaling, #2983S). The following secondary antibodies were used: Goat-anti-Rabbit IgG (H+L) Alexa Flour 488 (Invitrogen, A11088); Rabbit-anti-Mouse IgG (H+L) Alexa Fluor 568 (Invitrogen, A11031); Goat-anti-Rabbit IgG HRP Conjugated (Sigma-Aldrich, A0545); Rabbit-anti-Mouse IgG HRP Conjugated (Sigma-Aldrich, A9044).

## SUPPLEMENTARY MATERIAL



## Abbreviations

NaBut: sodium butyrate
TEM: transmission electron microscopy
MOMP: mitochondrial outer membrane permeabilization
mTOR: mammalian Target of Rapamycin
mTORC1: mammalian Target of Rapamycin Complex 1
ERK: extracellular signal-regulated kinase
MEK: mitogen-activated protein kinase kinase
SQSTM1: Sequestosome 1
LC3: microtubule-associated protein light chain 3
DMEM: Dulbecco's modified Eagle medium
FBS: fetal bovine serum
MTT: (3-(4,5-Dimethylthiazol-2-yl)-2,5-diphenyltetrazolium bromide
TMRM: Tetramethylrho-damine
SDS: sodium dodecyl sulphate
DMSO: dimethyl sulfoxide
BSA: bovine serum albumin
DAPI: 4,6-diamidino-2-phenylindole
TBS: Tris-buffered solution
CHAPS: 3-[(3-Cholamidopropyl) dimethylammonio]-1-propane-sulfonate hydrate
