# Supplementary material for: Targeted elimination of senescent Ras-transformed cells by suppression of MEK/ERK pathway
Source: Aging (Albany NY). 2017 Nov 14;9(11):2352–75. doi: 10.18632/aging.101325 (PMC5723691; doi:10.18632/aging.101325)
Supplement: Supplementary file 1 [file aging-09-2352-s001.pdf]

SUPPLEMENTARY MATERIAL

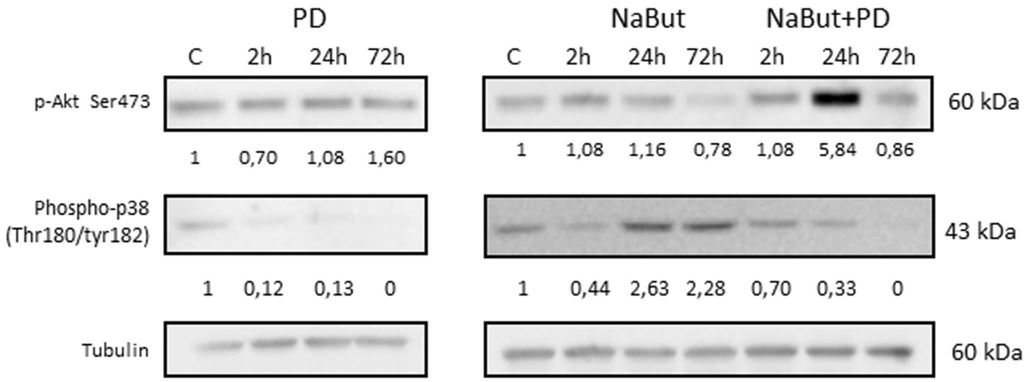

Supplementary Figure 1. MEK/ERK suppression decreases p38 MAPK phosphorylation, but does not change AKT (Ser473) phosphorylation. Western-blot analysis of AKT (Ser473) phosphorylation and p38 (Thr180/Tyr182) phosphorylation after treatment with inhibitors. Cells were cultivated for the indicated time and then lysed and processed to Western-blotting in 12% gel. Numbers below present densitometry of bands.
